# Level-2 visuo-spatial perspective-taking and interoception – More evidence for the embodiment of perspective-taking

**DOI:** 10.1371/journal.pone.0219005

**Published:** 2019-06-27

**Authors:** Thorsten Michael Erle

**Affiliations:** Department of Psychology, Social and Economic Cognition II, University of Cologne, Cologne, Germany; Anglia Ruskin University, UNITED KINGDOM

## Abstract

Level-2 visuo-spatial perspective-taking is an embodied process during which the perspective-taker mentally simulates a movement of his or her body into the location of the target. Evidence for the embodiment of this process so far exclusively stems from congruency effects in visuo-spatial perspective-taking experiments. Here, additional triangulation for the embodiment of this process is provided from an interindividual differences perspective. In a cross-sectional observational study, participants completed a behavioral level-2 visuo-spatial perspective-taking task and the heartbeat tracking task, which measures interoceptive accuracy and sensibility. Interoceptive accuracy is the objective ability to accurately perceive signals from within the body. In the present study, interoceptive accuracy was quantified by comparing the number of actual heartbeats observed via electrocardiographic recording to subjectively perceived heartbeats during that time. This measure was related to faster perspective-taking and better overall perspective-taking performance. Interoceptive sensibility refers to subjective beliefs about interoceptive abilities. Here, confidence in the estimated number of heartbeats served as a measure if interoceptive sensibility. Finally, the correspondence between interoceptive accuracy and sensibility is referred to as interoceptive awareness. Interoceptive sensibility and awareness were unrelated to perspective-taking. The study is a demonstration of the role interindividual differences in different facets of interoception play for embodied cognition. Implications for future research on links between embodied cognition and interoception are outlined and critically discussed.

## Introduction

Humans are uniquely adept at perceiving and representing the mental states of their conspecifics. This encompasses guesses about more elaborate intentions or beliefs of another person [1], but also of basic postures, gestures, or simple actions. One such basic process is visuo-spatial perspective-taking (VPT), the ability to mentally envision what is visible for another person (level-1 VPT) or how he or she perceives the world (level-2 VPT; [2]).

The mechanisms underlying VPT are well understood. Level-1 VPT is an egocentric process during which the perspective-taker mentally draws a line between the eyes of a person and an object. The difficulty of this process is determined by the distance between these two and the process does not entail any embodiment [3]. Level-2 VPT is an embodied process during which the perspective-taker mentally simulates a body movement into the physical location of a target [4]. The difficulty of this operation hinges on the angular disparity between perspective-taker and target and level-2 VPT only occurs when the reference frames of perspective-taker and target mismatch [3–4].

But how is this process embodied and which evidence supports this assertion? Embodied cognition frameworks assume human cognitions to be grounded in the motor and perceptual systems of the body [5–6]. In this vein, the simulation of level-2 VPT relies on the representation of one’s body schema in space and a simulated movement to another physical location [4]. Supporting this idea, rotating participants’ bodies prior to VPT towards/away from a target reduced/enhanced VPT reaction times (RTs; [4]). Such congruency effects show that during VPT, people indeed “mentally move” their body and the movement distance determines how long the process takes. Although the embodiment of level-2 VPT is a highly robust phenomenon that has been replicated many times [3–4, 7–12], the first goal of this article is to provide evidence for the embodiment of level-2 VPT not based on such congruency effects, but from an interindividual differences perspective. This is important as embodied cognition research sometimes tends to neglect interindividual differences [13]. Notably, in the case of visuo-spatial perspective-taking, there are studies showing that the embodiment of level-2 visuo-spatial perspective-taking indeed differs between specific populations [14–15]. These studies showed that especially women and participants high in social skills (as measured by the Autism Quotient, [16]) show stronger embodiment of visuo-spatial perspective-taking, which is in line with the view that visuo-spatial and higher forms of perspective-taking (i.e., empathic perspective-taking) are related [7–10].

Another interindividual difference that has not yet been linked to level-2 VPT but that is very closely related to the idea of embodied cognition, is interoception (note that previous research has investigated links between level-1 VPT and interoception, see [17]). Interoception is the ability to perceive signals from the bodily interior such as the heartbeat, breathing, or gastrointestinal signals (for reviews, see [18–20]). Recently, a model of interoception was proposed according to which interoception encompasses the dimensions of interoceptive accuracy (IAcc), interoceptive sensibility (IS) and interoceptive awareness (IAw) [21]. IAcc is the actual ability to perceive bodily signals (assessed, e.g., with the heartbeat tracking task (HTT), [22]). IS refers to a person’s subjective beliefs about his or her IAcc, and IAw is the correspondence between IAcc and IS.

Indeed, various embodiment phenomena such as the rubber-hand illusion [23–24], the embodiment of weight ([25]; Exp. 1), or softness in value judgments ([25]; Exp. 2), as well as the experience of physical effort more generally [26] have been related to interoception (for a review, see [27]). Although many studies suggest stronger embodiment for participants scoring high on various indicators of interoception, it is noteworthy that they targeted different embodiment phenomena and used different measures of interoception such as the heartbeat-discrimination task [28] (used in [23]), the HTT [24], [25]; Exp. 2, both as measures of IAcc or the Private Body Consciousness Scale (PBC; [29]; used in [25]; Exp. 1), which is a measure of IS. Given novel insights into the tripartite nature of interoception [21], researchers should be advised to more thoroughly specify which facet of interoception is related to which embodiment phenomenon by which mechanism. The present article’s second goal therefore is to extend these findings to level-2 VPT as another embodied process and to precisely investigate which facets of interoception are related to the embodiment of level-2 VPT.

Being strongly attuned to signals from the body means that simulations of owning, moving, and transforming one’s body schema are facilitated in two ways. First, accurate interoception provides a head-start for the simulation as individuals with high IAcc will judge the body’s initial state more quickly and accurately. Second, once the simulation has concluded, the generation of the anticipated end-state will also benefit in the same way. In terms of performance, level-2 VPT should be completed faster and more accurately by individuals scoring high on IAcc, and hence a positive relation between IAcc and VPT ability was predicted in the present study.

Importantly, only IAcc should be related to stronger embodiment as only objectively correct perceptions of (signals from within) the body can increase the accuracy of simulations. For IS and IAw, it is unclear how this would relate to more strongly embodied cognition as previous research has shown that IAcc and IS are not necessarily correlated [21], implying that IS is not necessarily an accurate view of one’s interoception. Similarly, being highly aware of one’s interoceptive abilities (IAw) does not change actual interoceptive performance, that is, IAcc. In the end, only how well one is able to judge the body’s state accurately will determine how much any embodied simulation is sped up or slowed down. To clearly delineate whether IAcc, IS, or IAw contribute to better embodied simulations (cf. [23–25]), in the present study IAcc and IS were concurrently assessed and IAw was computed based on their correspondence. Faster and more accurate VPT was predicted only for participants with high IAcc whereas for IS and IAw no relation to embodiment was predicted as it is unclear whether subjective beliefs about interoception or the awareness of one’s ability to perceive the body have an effect on actual embodied processing.

## Materials and methods

### Sample size determination and open practices

Data and materials for this study can be found at https://osf.io/gezdy/. To be able to detect a medium-sized correlation (*r* = .30) with a statistical power of (1-β) = .80 in a two-tailed test, a sample of at least *N* = 82 participants was needed. *N* = 101 participants were tested, but due to technical difficulties electrocardiographic (ECG) data for *n* = 4 individuals were lost and *n* = 1 participant was removed from all analyses due to unrealistic reporting of heartbeats (>400/minute). Finally, for *n* = 10 participants no IAw score could be computed as these participants always indicated the same level of IS and hence, IAw (i.e., the correlation between IS and IAcc) could not be computed for these participants. The final sample size thus was *N* = 96 for all analyses except those including IAw (*N* = 86), slightly surpassing the goal.

### Procedure

Participants were tested individually in 90-minute sessions. This 15-minute study was run first in a battery followed by unrelated experiments ([30]; Exp. 4). After providing signed informed consent, participants completed the VPT task, the HTT, and provided demographic data. After all tasks were completed, participants were thanked, debriefed upon request, and received a compensation of 10 €. The study was approved by the local ethics committee at the University of Würzburg and was conducted according to the principles expressed in the Declaration of Helsinki.

### Measures of VPT

**Fig 1:**
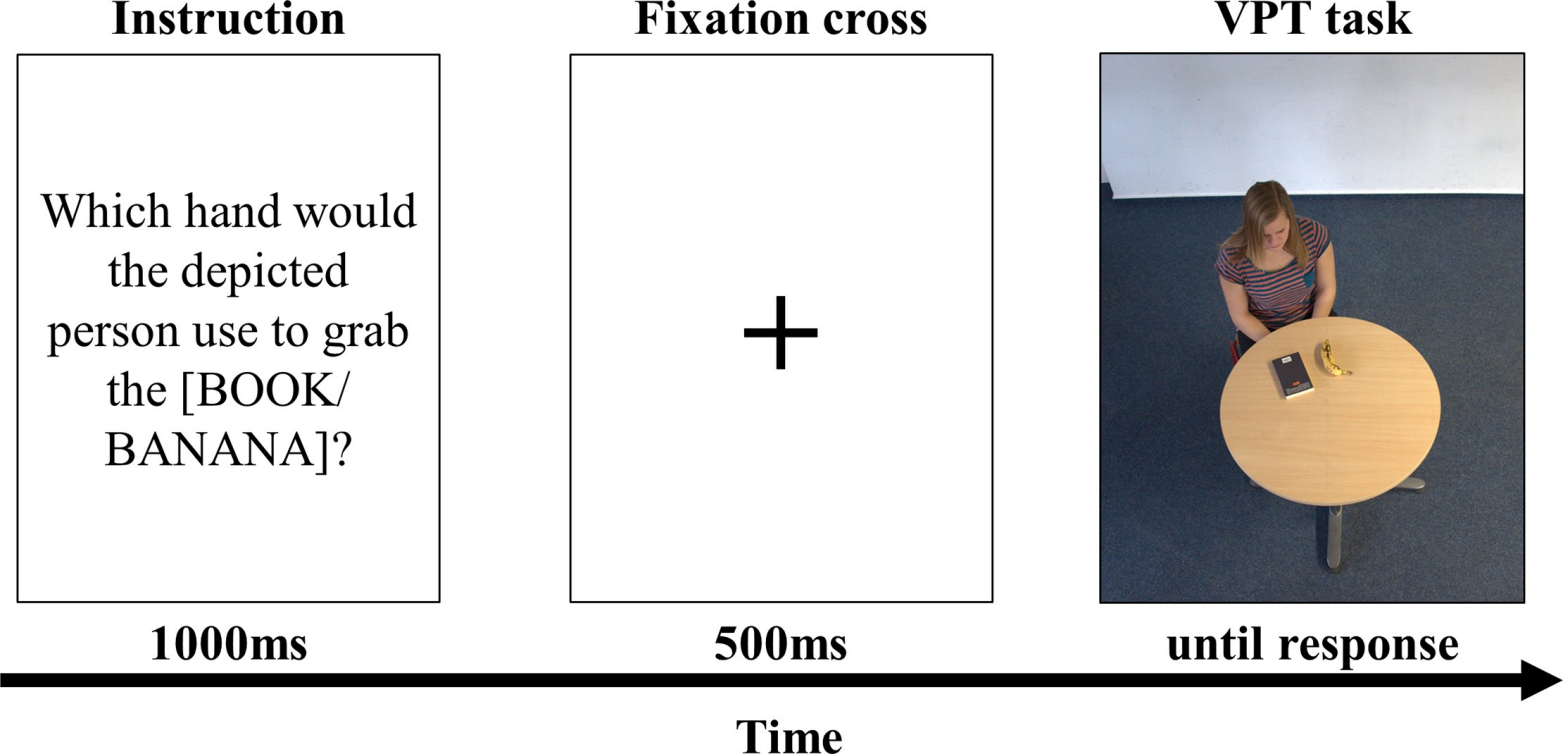
Exemplary trial sequence of the VPT task.

As a measure of VPT, a computerized VPT task [4] was adopted using openly available stimuli [10]. During every trial, participants see a person (the “target”) sitting at a table with two objects on the computer screen. Their task is to “grab” one of the objects from the target’s perspective, using two response keys, in this case the two CTRL keys on a standard keyboard. Crucially, the angular disparity between participant and target is manipulated so that the target sits at an angle of 0°, 40°, 80°, 120°, or 160° (clockwise or counterclockwise) from the participant. The right CTRL key indicated that the target would use his or her right hand to grab the instructed object and the left CTRL key corresponded to the target’s left hand. An exemplary trial sequence is depicted in Fig 1.

For lower angular disparities (0°–40°), previous research has shown that no embodied simulation occurs [3–4, 11–12], because here the spatial relations of the scene are identical from the participant’s egocentric and the target’s perspective. Contrary to this, starting at 80° of angular disparity the participant’s egocentric frame of reference and that of the target start to differ. To overcome this discrepancy, VPT and hence an embodied simulation occurs.

Participants completed 80 trials of this task (16 at each level of angular disparity). The target object, the target person’s rotation direction (clockwise or counterclockwise), and the arrangement of the two objects were counterbalanced across the trials of every level of angular disparity. The sequence of the 80 trials was randomized anew for every participant.

Three dependent measures of VPT performance were computed. (1) For correct responses, RTs of egocentric trials were subtracted from perspective-taking trials as a measure of VPT speed, with higher numbers indicating slower VPT. (2) The percentage of errors during egocentric trials was subtracted from perspective-taking trials as a measure of VPT accuracy, with higher numbers indicating less accurate VPT. (3) As RT analyses exclusively focus on trials during which the participant responded correctly whereas error analyses focus exclusively on trials where the opposite was the case, a third measure was computed to analyze all available data conjointly, conserving the highest amount of information possible. As both speed and accuracy are indicators of how well a person completed the VPT task, but the two dependent measures have different metrics, for this analysis both measures were *z*-standardized to be in the same metric and these two scores were then averaged into an overall performance index of VPT with higher numbers indicating worse performance.

### Measures of interoception

To measure participants’ interoception, the HTT [22] was used, which measures participants’ ability to accurately perceive their heartbeat. This task has the advantage that it is a behavioral, objective, and performance-based measure of IAcc that also includes an assessment of IS. Finally, the HTT has been used in previous works relating interoception to embodied cognition (e.g., [23–25]).

During the HTT, participants are asked to calmly sit while feeling and silently counting their heartbeats for a given time without checking their pulse. Since previous research has shown that HTT performance can be influenced by beliefs about heart rate and time perception [31–33], it is important to note that we employed what previous researchers have labeled the “standard instruction” for the HTT, which asks participants simply to “count all heartbeats that you feel in the body” ([34]; the exact instructions used in the present study can be found at https://osf.io/gezdy/). Participants first complete a training trial of 10 seconds to familiarize them with the procedure, followed by four test trials (25s, 35s, 45s and 60s). After every interval, participants report their counted heartbeats. Concurrently, participants’ actual number of heartbeats is recorded via ECG. Participants never received feedback about their performance. In the present study, ECG signals were acquired using a 16-channel amplifier (V-Amp, Brain Products GmbH, Gilching, Germany) with a sampling rate of 1000Hz. To calculate IAcc, this formula was used:

$$IAcc=\frac{1}{4}*\sum (1-\frac{\left|recorded\;heartbeats-counted\;heartbeats\right|}{recorded\;heartbeats})$$

As a measure of IS, participants reported their confidence in their HTT performance on a ten-point scale after every interval. The measure of IS was the average of these ratings. Finally, correlations between participants’ IAcc and IS served as an index of IAw [21].

### Sample

A convenience sample of *N* = 101 participants (*n* = 82 female; *n* = 18 male; *n* = 1 missing data; age: *M* = 24.60, *SD* = 4.59) from the Würzburg area completed the study. Although participation was open to everyone, the sample predominantly consisted of students (*n* = 76). The mean body mass index (BMI) of the sample was *M* = 23.01 (*SD* = 3.90), their mean IAcc was *M* = .67 (*SD* = 0.17), and their mean IS was *M* = 4.31 (*SD* = 1.97).

## Results

**Fig 2:**
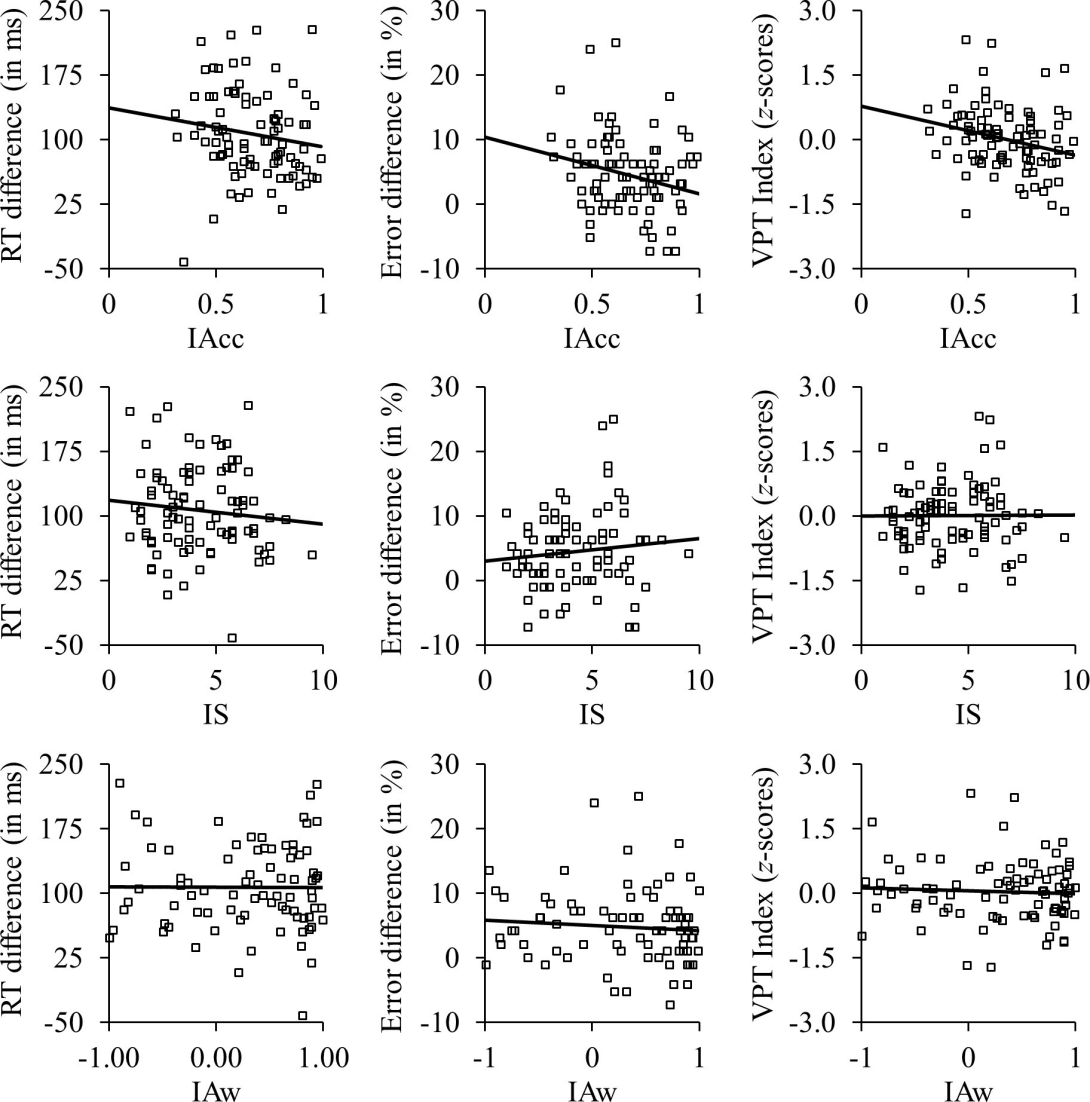
Correlations between interoception and VPT. Raw data and correlations between IAcc (top row), IS (middle row), and IAw (bottom row) with VPT speed (left column), accuracy (middle column), and overall performance (right column).

Since three tests were computed for IAcc, IS, and IAw, the significance level was set to α = .05/3 = $.01\bar{6}$. Since for IAcc, IS, and VPT RTs the assumption of normality was violated (all *d*_*96*_ > 0.09, all *p*s ≤ .049), but all variables were metric, Kendal’s τ correlation coefficients and *R^2^* as a measure of effect size are reported following previous suggestions [35]. For non-significant correlations, also Bayes Factors quantifying the support for the null-hypothesis are reported. BMI was uncorrelated with IAcc, τ(94) = -.12, *p* = .095, and therefore not included as a covariate. Heart rate, on the other hand, was correlated with IAcc, τ(94) = -.29, *p* < .001, and therefore partial correlations controlling for heart rate are reported. Fig 2 shows the raw data underlying all analyses.

### VPT speed

As expected, on average participants reacted slower during VPT than during egocentric trials (difference: *M* = 108.26 ms, *SD* = 52.32, range: -42.02–227.97). There was a negative correlation between the VPT RT difference between egocentric and perspective-taking trials and IAcc, τ(94) = -.16, *p* = .014, *R^2^* = .06, indicating that participants with higher IAcc were faster at solving the VPT task. There was neither a significant correlation between IS and VPT, τ(94) = -.04, *p* = .589, *R^2^* < .01, *BF*_*01*_ = 6.19, nor between IAw and VPT, τ(84) < .01, *p* = .967, *R^2^* < .01, *BF*_*01*_ = 7.11. These Bayes Factors mean that the data were about 6/7 times more likely under the assumption that there is no correlation between IS/IAw and VPT speed, which is conventionally [36] interpreted as “moderate evidence” in favor of in this case the null-hypothesis.

### VPT accuracy

As expected, on average participants committed more errors during VPT than during egocentric trials (difference: *M* = 4.32 ms, *SD* = 6.18, range: -10.42–25.00). For the error difference between egocentric and perspective-taking trials, there was a neither a significant correlation with IAcc, τ(94) = -.13, *p* = .047, *R^2^* = .04, *BF*_*01*_ = 0.91, nor IS, τ(94) = .05, *p* = .480, *R^2^* = .01, *BF*_*01*_ = 6.01, nor with IAw, τ(84) = -.11, *p* = .154, *R^2^* = .03, *BF*_*01*_ = 2.51, indicating that VPT accuracy was independent of interoception. While the evidence was completely even for IAcc, the data were about 6/3 times more likely under the assumption that there is no correlation between IS/IAw and VPT accuracy, meaning the evidence against a correlation was “barely worth a mention” (IAw) or “moderate” (IS), respectively [36]. Thus, the present data are inconclusive about the existence of a correlation between IAcc/IAw and VPT accuracy and suggest that VPT accuracy is independent of IS. It should be noted, however, that the ability to detect significant effects was limited as participants committed only very few errors (*M* = 6.09 [%], *SD* = 4.09) in general.

### Overall VPT performance

There was a negative correlation between the overall VPT performance index and IAcc, τ(94) = -.18, *p* = .006, *R^2^* = .08, indicating that participants with higher IAcc were generally better at solving the VPT task. There was neither a significant correlation with IS, τ(94) < .01, *p* = .959, *R^2^* < .01, *BF*_*01*_ = 7.66, nor with IAw, τ(84) = -.05, *p* = .490, *R^2^* < .01, *BF*_*01*_ = 5.62. The data were about 8/6 times more likely under the assumption that there is no correlation between IS/IAw and overall VPT performance (“moderate evidence”; [36]).

## Discussion

This is a novel demonstration of how interindividual differences, and interoception specifically, affect embodied processing. Whereas IAcc correlated significantly with VPT speed and overall performance, no significant correlation for VPT accuracy was observed. Thus, concerning the article’s first goal, it provides additional evidence for the embodiment of level-2 VPT with at least some consistency. For IS and IAw, on the other hand, no significant relations to level-2 VPT were observed and there was mostly “moderate evidence” [36] against a relation between IS/IAw and VPT. This dissociation was generally in line with the second goal of the study, that is, showing that an important moderator of the interoception-embodiment relation is the way in which one measures these constructs: Beliefs about interoceptive capabilities (i.e., IS and IAw) have no bearing on embodied processing. The ability with which we can perform embodied simulations benefits from our actual ability to perceive the body (IAcc), and neither from how well we believe we can perceive it (IS), nor from how aware we are that we can(not) perceive it (IAw).

Although the results largely supported the predicted association between IAcc and VPT, the absence of a correlation with VPT accuracy needs an explanation. First, it is possible that IAcc is only related to VPT speed, but not accuracy. However, as there are usually no speed-accuracy-tradeoffs in level-2 VPT tasks [3–4; 11–12], there is no theoretical reason to assume this. Second, it is possible that the ability to detect a significant correlation was limited by the overall low number of errors made on the VPT task. Finally, the non-significant correlation could represent a false-negative finding. Indeed, while the *p*-value for this test was not statistically significant (*p* = .047) against the adjusted significance criterion, the correlation was indistinguishable from the correlations between IAcc and VPT speed and overall performance (both *Z*s < 0.50, both *p*s > .624), suggesting that this relation should be revisited in future research.

Furthermore, contrary to the present results, previous research has found significant relations between IS and embodiment ([25]; Exp. 1). However, previous studies measured IS using the PBC [29], while in the present study participants’ confidence in their HTT performance served as a measure of IS. Although the PBC matches today’s definition of IS, it might alternatively be seen as a self-report measure of IAcc. To definitively locate the PBC within either IAcc or IS, future works should replicate the present study with an additional assessment of the PBC to see whether it follows the pattern of IAcc or IS measures (see also [37]). In any case, the usefulness of the PBC or the presently used confidence measure of IS (and thus also IAw) in embodiment research is debatable given the limitations of self-reports and the above-mentioned role of beliefs for embodied simulations.

Regarding the assumed embodiment of level-2 VPT it is important to note that while the present results are in line with theoretical predictions of an embodied cognition account, additional experimental evidence is needed to corroborate this. One limitation of using a level-2 VPT task without actively manipulating participants’ body posture is that RT differences between egocentric and perspective-taking trials serve only as a proxy for embodiment. While previous research has shown that an embodied self-transformation is a common or even predominant strategy during this task [9], the large dispersion of RT differences (with some participants even showing negative RT and error differences) indicates that this need not be the way all participants solve the task.

For example, previous research has shown that level-1 VPT paradigms can be solved by multiple means and has proposed a two-dimensional framework of how level-1 VPT is achieved [38]. In this framework, the two dimensions underlying level-1 VPT are conflict handling (resolving VPT conflicts) and attentional focus (default of the self or the other). It would be important to explore these dimensions for level-2 VPT as well. Several interesting predictions could be derived from this framework: First, participants high on conflict handling and highly other-centered participants should be faster at level-2 VPT tasks, too. Second, as it relates to interoception it is debatable whether these basic cognitive profiles are related to embodiment or not. The present results suggest that there might be a relation between cognitive predispositions, interoception, and embodiment. Potentially, IAcc is an important mediator between these dimensions [38] and embodied processing, but this would have to be confirmed in future research.

Another framework that the present data are compatible with is that of interoceptive inference [39], an extension of predictive coding [40] to interoception. Predictive coding means that perception is guided by constant predictions about the environment that are updated online in comparison to actual experiences [39–40]. The predictive system strives to minimize prediction errors by either updating the predictive model of the world or by performing actions that more closely adhere to the predictive model. According to this view, greater IAcc provides a more accurate perception of the body in its environment and a stronger experience of body ownership [41]. Higher IAcc thus reduces the number of predictive errors and feedback loops associated with such errors and might enable the perceiver to more efficiently and successfully generate behaviors that match the predictive network [39]. In line with the present findings, an interoceptive inference account would predict that only IAcc is related to stronger embodiment as only objectively correct perceptions of bodily signals can increase the accuracy of predictive network, while for IS and IAw there should be no relation. Thus, interoceptive inference could largely be arranged with the embodiment of level-2 VPT. It must be noted, however, that in a behavioral study such as the present one, predictive coding is only one possible post-hoc explanation of the results that must be verified in studies concurrently assessing patterns of brain activation.

Finally, while this study has major methodological strengths (e.g., focus on a well-established and replicable embodiment effect, use of objective and behavioral measures), it also has several limitations. A first limitation is the statistical power of the present study (observed power to detect the mean IAcc correlation in a one-/two-tailed test: (1-β) = .82/.72). Therefore, a (pre-registered) direct replication with a larger sample size or a directional prediction (*N* = 92/117 to achieve (1-β) = .80 in a one-/two-tailed test) is warranted, also to provide stronger evidence (*BF*_*01*_ > 10) against potential relations between IS/IAw and VPT.

Second, the correlational design of this study does not allow for claims about causality. While it is intuitively appealing to think that people with great interoceptive abilities should be predisposed to stronger embodied processing, the opposite could be true as well. To answer this question, studies that longitudinally look at preferably multiple embodiment effects in participants who train their interoceptive abilities (e.g., via breathing mediation or body scan exercises) are needed.

Third, recently some publications have raised doubts about the validity of the HTT as a measure of IAcc [34] and other researchers have pointed out that high scores on the HTT could be influenced by factors independent of interoception such as one’s fitness or resting heart rate [42]. In the present study, BMI was unrelated to HTT performance, and the present findings were observed while controlling for heart rate. Nonetheless, future research should conceptually replicate the present results with different measures of IAcc [43–44] to further corroborate whether the present relation is reliable and valid.

Despite these limitations, the present report provides novel evidence for the embodiment of level-2 VPT from an interindividual differences perspective. The results should inspire further research on the role of the different facets of interoception in embodied cognition, which is a largely neglected yet promising research area.
